# Periscope

**Published:** 1881-12

**Authors:** 


### PERISCOPE.

Bibliotheque Homœopathique, May :—Dr. Chancerel reports a case of abscess of left iliac fossa following an attack of puerperal peritonitis. The patient had severe pains in left inguinal region which compelled her to lie upon the back. A tumor appeared in this region which was exquisitely sensitive to the touch. *Hepar Sulphur* 30 was given, then *Silicea* 30, then *Hepar* 30, then *Asafœtida* 30, and finally *Causticum* 30, which latter promptly cured. The doctor then gives the indications for *Causticum* according to Bœnninghausen. Dr. Duprat reports a case of uterine hemorrhage from the presence of a polypus. The patient had pains in the back, sense of weight in lower abdomen, sensation of something about to escape from the vulva, and irresistible desire to urinate. *Crocus sat.* 6th was administered, then later on *Calc. carb.* 200, then *Thuja* 18. The result was the polypus came away spontaneously. The same physician also relates a case of mucous polypus in the nose, with continual coryza, corrosive nasal discharge of greenish color and disagreeable odor, warts on the hands. *Thuja* 6th caused the polypus to come away without surgical operation. Dr. Serrand cured a case of neuralgia of internal saphenous nerve of right leg, where the *patient* was better from walking and worse from being perfectly quiet, with *Rhus tox.*, followed by *Hamamelis virgin.* 6th, internally, and a weak dilution of *Hamamelis* externally.

Dr. Duprat relates a case of chorea accompanied by anemia and vermicular symptoms. The indications were: *epileptiform fits; involuntary jerking motions of left arm and shoulder; low spirited; spasmodic laughter followed by crying; weakness of mind; leucorrhœa and pruritis of vulva; ascarides; constipation and diarrhœa; sour risings from the stomach. Calc. carb.* was accordingly given, followed later by *Puls.* In two weeks everything was better. Numbers of worms were discharged, which induced the doctor to give *Cina.* This last was, we think, an unwise prescription, since the appearance of the worms was due to the curative action of the preceding medicines, wherefore they should not have been disturbed in their action. However, she was restored to health which "seemed brilliant and enduring."

Dr. Chanceral translates from the Spanish a case of lead-poisoning having the following symptoms: pale face; also yellowness of skin, and conjunctiva; mental agitation; sleeplessness; pulse slow; sadness; pressing headache; bitter taste; lead-line on gums; intense crampy pains from the umbilicus to pubis; constipation; vomiting of green bile; nightly aggravations. *Nux vom.* 30th followed by *Ipecac.* 200, then *Bell.* 200, then *Lyc.* 30 cured him. From the same source is reported a case of ulceration of nearly the whole of the lower third of the external surface of the right leg with *burning pains, paleness of bottom of ulcer, abundant ichorous suppuration.* For these symptoms *Arsenic* was prescribed, which cured promptly. A case of pleurodynia from taking cold exhibited the following symptoms: *lancinating pain in lower right chest, worse from motion, cough and inspiration.* *Bryonia* 12 was finally given after the mistaken administration of *Arnica* and *Mercurius.* The remedy produced an immediate cure, at the same time ejecting a large worm. A case of persistent cough, worse from hunger; the least movement fatigued him and caused perspiration; *Bryonia* was given, curing him and causing expulsion of a large tape-worm.

HOMŒOPATHIC WORLD, JULY:—Dr. Cooper reports two cases of perforation of ear-drum *cured*, the one with *Terebinth.* 1st and then 200; the other with *Hepar S.* 3, and later 200. No indications are given, hence these cases are not very instructive. They are noticed, however, because of the fact that drug treatment in potency *repaired* a perforated ear-drum and restored the hearing.

BRITISH JOURNAL OF HOMŒOPATHY,* JULY:—Dr. Ussher reports a case of a type-setter who had *tingling sensation in scrotum with feeling of discomfort; sensation of fluttering in heart and throat.* These are claimed by the doctor as indications for *Natrum mur.*, which was given in 6th and later the 30th dilution with the effect of curing the patient.

MEDICAL COUNSELOR, JULY:—Dr. Wigg reports the case of a lady who seemed about to miscarry. She complained of pain in the back as if it would fall to pieces; expulsive pains; and flooding. *Belladonna* 6 was given, and in an hour there were expelled from the uterus "ten pounds of hydatids, in number about sixteen thousand."

HAHNEMANNIAN MONTHLY, AUG.:—Dr. McGuire relates three cases. One of them a lady, had been subject to *epileptiform convulsions, headache and dysmenorrhœa.* She suddenly lost the sight of

---

*We regret that our English exchanges have not been received lately.

the eye followed by severe *headache, unconsciousness, vomiting* and appearance of balls of fire before the eyes. The eye trouble was diagnosed as choroiditis circumscripta. These troubles were much aggravated at the menstrual period. The different remedies selected failed to produce any effect. *Verat. vir.*, tincture, was then given, producing immediate relief of pains, and later on potencies of the same drug were administered, producing a complete restoration of vision. It is to be observed that the *instructive* value of this case is somewhat marred by several intercurrent remedies which, of course, create doubt as to the part played by by *Verat. vir.*, ultimately. The second case was a lady who had been subjected to acute metritic cellulitis. Then came on failure of vision, dull pain in the eye, and heavy pain in left side of head, better from pressure, worse from lying down. Diagnosis of eye trouble was "congestion papille." *Verat. vir.* 1st cured in five weeks. In the third case the patient became "suddenly blind." *Verat. vir.* was given and promptly cured.

Dr. Allen reports a case of prosopalgia left side. Symptoms were: intense pain, with inability to eat solid food on account of *aggravation from moving* the jaws; *worse also from washing or touching the part*—even touching the ends of the moustache increased the pains; worse after stool and *from exposure to cold winds. Had taken much purgative medicine; drowsy and tired in morning; aversion to getting up in morning. Nux. V.* 200 produced much improvement. *Mezereum* 6 was then given which cured him, completely, after severe suffering for ten years.

Dr. Perkins reports a case of traumatic gangrene of the hand to which was superadded phlegmonous erysipelas, for which *Rhus tox.* 3, was given which prevented the extension of the erysipelas, and enabled the doctor to perform successful amputation. W. M. J.

